# Identification and Validation of Reference Genes in *Clostridium beijerinckii* NRRL B-598 for RT-qPCR Using RNA-Seq Data

**DOI:** 10.3389/fmicb.2021.640054

**Published:** 2021-03-18

**Authors:** Katerina Jureckova, Hana Raschmanova, Jan Kolek, Maryna Vasylkivska, Barbora Branska, Petra Patakova, Ivo Provaznik, Karel Sedlar

**Affiliations:** ^1^Department of Biomedical Engineering, Faculty of Electrical Engineering and Communication, Brno University of Technology, Brno, Czechia; ^2^Department of Biotechnology, University of Chemistry and Technology Prague, Prague, Czechia

**Keywords:** HKG, housekeeping genes, non-model organisms, biofuel, *Clostridium*

## Abstract

Gene expression analysis through reverse transcription-quantitative real-time polymerase chain reaction (RT-qPCR) depends on correct data normalization by reference genes with stable expression. Although *Clostridium beijerinckii* NRRL B-598 is a promising Gram-positive bacterium for the industrial production of biobutanol, validated reference genes have not yet been reported. In this study, we selected 160 genes with stable expression based on an RNA sequencing (RNA-Seq) data analysis, and among them, seven genes (*zmp*, *rpoB1*, *rsmB*, *greA*, *rpoB2*, *topB2*, and *rimO*) were selected for experimental validation by RT-qPCR and gene ontology (GO) enrichment analysis. According to statistical analyses, *zmp* and *greA* were the most stable and suitable reference genes for RT-qPCR normalization. Furthermore, our methodology can be useful for selection of the reference genes in other strains of *C. beijerinckii* and it also suggests that the RNA-Seq data can be used for the initial selection of novel reference genes, however, their validation is required.

## Introduction

Reverse transcriptase-quantitative real-time polymerase chain reaction (RT-qPCR) is the most commonly used technique to quantify gene expression due to its high sensitivity, specificity, and reproducibility (Ginzinger, 2002). Correct quantification of mRNA relies on data normalization that removes differences in the extraction yield, reverse-transcriptase activity and efficiency of PCR amplification (Vandesompele et al., 2002; Bustin et al., 2009). The most commonly used normalization method utilizes the so-called reference genes against which are gene expression data relatively quantified. Reference genes should maintain a constant mRNA expression regardless of the experimental conditions, different tissues, cells, or life cell phases (Derveaux et al., 2010). Thus, correct selection plays a crucial role in accurate data normalization. However, there is no universal reference gene and many studies have shown that the expression of commonly used reference genes is not always stable (Liang et al., 2014; Chapman and Waldenström, 2015; Jo et al., 2019), and reference genes should be selected individually for each organism and experimental condition (Liang et al., 2014). Furthermore, literature mining is not an appropriate approach for their selection and cannot be used in most cases. On the other hand, recent studies use RNA sequencing (RNA-Seq) technology for the evaluation of the whole transcriptomes to find novel candidates for reference genes (Carvalho et al., 2014; Hu et al., 2016; Pombo et al., 2017).

Thus far, suitable reference genes have been determined in different *Clostridium* species, such as *Clostridium difficile* (Metcalf et al., 2010), *Clostridium botulinum* (Kirk et al., 2014), and *Clostridium ljungdahlii* (Liu et al., 2013). In *Clostridium beijerinckii* NCIMB 8052, 177 putative housekeeping genes were previously identified based on transcriptomic data (Wang et al., 2011). However, thus far, no evaluation study of reference genes has been performed for this species. *C. beijerinckii* has been found to be a promising microorganism for industrial production of biobutanol, and efforts to increase butanol productivity by means of genetic and metabolic engineering of its strains have been recently reported (Agu et al., 2019; Xin et al., 2020). However, the strain engineering of *C. beijerinckii* is hindered by insufficient understanding of cellular physiology and regulatory mechanisms of gene expression. Despite the recent progress in CRISPR-associated methods of genetic engineering tailored to *C. beijerinckii* (Wang et al., 2016), the identification of valid endogenous reference genes for RT-qPCR is lacking.

In the present study, we selected seven putative reference genes (*zmp*, *rpoB1*, *rsmB*, *greA*, *rpoB2*, *topB2*, and *rimO*) for *C. beijerinckii* NRRL B-598 based on the RNA-Seq data that were obtained under different experimental conditions and at different time-points. The seven candidate reference genes were described and summarized by the gene ontology (GO) enrichment analysis and further tested for expression stability by RT-qPCR experiments and evaluated by four statistical algorithms: NormFinder (Andersen et al., 2004), RefFinder (Xie et al., 2012), Coefficient of variation (CV) analysis (Boda et al., 2009), and Pairwise ΔCt method (Silver et al., 2006). According to the stability rating, we identified a novel set of reference genes that can be used for the normalization of RT-qPCR data of *C. beijerinckii* NRRL B-598. As the strain *C. beijerinckii* NRRL B-598 shares high homology with other *C. beijerinckii* strains (Sedlar et al., 2017), the results will be useful for all strains of the species.

## Materials and Methods

### RNA-Seq Data Pre-processing

RNA sequencing data were obtained in our previous studies in which we first observed the transcription changes during standard ABE fermentation of *C. beijerinckii* (Sedlar et al., 2018; Patakova et al., 2019). The RNA-Seq data consisted of five replicates and six samples/time-points per replicate. Next, we evaluated the transcriptional response of *C. beijerinckii* to butanol shock (Sedlar et al., 2019) with two replicates and six samples/time-points each. Together, the available RNA-Seq transcriptomes consisted of 42 samples collected across 12 diverse time-points and conditions (see Table 1).

**TABLE 1:** RNA-Seq samples.

Sample name	Number of replicates	Cultivation conditions	Time-point (h of cultivation)	Sample description	References
T1	5	Bioreactor cultivation, TYA medium containing glucose	3.5	Taken in the middle of acidogenic phase when OD 600 nm reached approximately 1	Sedlar et al., 2018; Patakova et al., 2019; Vasylkivska et al., 2019
T6			23	Taken during solventogenic phase, when first mature spores were observed	

T_b_0	2	Bioreactor cultivation, TYA medium containing glucose, butanol addition (at 6 h)	6	Taken directly before butanol addition, close to shift between acidogenesis and solventogenesis	Sedlar et al., 2019; Patakova et al., 2020
T_b_2			7	Taken 1 h after approximately 4.5 g/L (non-lethal concentration) of butanol was added to the medium to capture short-term response to the shock	

Pre-processing of the RNA-Seq data was performed in the same manner as in our previous studies. However, the analysis was recalculated to ensure the utilization of the same versions of particular tools for all samples. The data quality assessment was conducted by FastQC (v0.11.5) and summarized reports across samples were generated by MultiQC (v1.7) (Ewels et al., 2016). Trimmomatic software (v1.36) (Bolger et al., 2014) was used for quality and adapter trimming. The RNA-Seq reads corresponding to 16S and 23S rRNA genes sequences were filtered out by SortMeRNA (v2.1) (Kopylova et al., 2012) and SILVA database (Quast et al., 2013) (v132). Cleansed reads were mapped by STAR (v 2.7.3a) (Dobin et al., 2013) to a reference genome of *C. beijerinckii* available in the GenBank database under accession CP011966.3. Mapping results in SAM (Sequence Read Alignment/Map) file format were indexed and converted to BAM (Binary Read Alignment/Map) files by SAM-tools (v1.7) (Li et al., 2009).

Mapped reads were counted by the featureCounts function from R/Bioconductor Rsubread (v2.2.6) package (Liao et al., 2014, 2019), utilizing two counting strategies: one for uniquely mapped reads and the other for multi-mapping reads. Raw count tables were further processed in R (v4.0.2), using custom-made scripts, and then used for estimation of TPM (transcript per million) values for each gene and sample. From obtained TPM values, we determined the mean value and CV [standard deviation (SD) to the mean (μ)] of TPM values for each gene.

Furthermore, we performed differential expression analysis on raw count tables using R/Bioconductor DESeq2 (v1.28.1) package (Love et al., 2014) between all 12 time-points. A GO enrichment analysis was conducted by the R/Bioconductor topGO (v2.40.0) package (Alexa and Rahnenfurher, 2020) based on the *C. beijerinckii* GO annotation map created in our previous study (Sedlar et al., 2019).

### Selection of Candidate Reference Genes

The selection of candidate reference genes was conducted by a series of filtering steps according to the results of processed RNA-Seq data: TPM values, CV of TPM values and results from differential expression analysis. Based on the results from differential expression analysis between all 66 time-points pairs, we counted the number of times each gene was not significantly regulated, *p*-adjust value > 0.1 (Benjamini–Hochberg correction) and filtered out all genes that did not pass the threshold of 50 insignificant changes. In the next step, we eliminated genes with a mean TPM value lower or equal to 35 TPM. Finally, we removed genes with the CV of TPM greater or equal to 30%. After each data filtering step, we compared results from both counting methods (unique and multi-mapping options), and only the genes reported by both methods were preserved for further processing.

### Samples for RT-qPCR

The gene expression of the seven candidate reference genes *zmp*, *rpoB1*, *rsmB*, *greA*, *rpoB2*, *topB2*, and *rimO* were assessed in the following cultivation samples: T1, T6, T_*b*_0, T_*b*_2, t0, t1, and t1_CH (see Supplementary Material 1). The origin of the samples T1, T6, T_*b*_0, and T_*b*_2 is described in our previous transcriptomic studies (Sedlar et al., 2018, 2019) (see Table 1). Briefly, samples T1 and T6 were obtained during a bioreactor batch cultivation of *C. beijerinckii* NRRL B-598 on the TYA medium at time points of 3.5 and 23 h, respectively (Sedlar et al., 2018). Samples T_*b*_0 and T_*b*_2 were obtained during bioreactor cultivation of *C. beijerinckii* NRRL B-598 on a TYA medium with added butanol at time points 0 and 1 h after butanol addition, respectively (Sedlar et al., 2019). The samples t0, t1, and t1_CH were obtained as follows: 480 ml of the TYA medium was inoculated with a spore suspension of *C. beijerinckii* NRRL B-598 and cultured overnight at 37°C in 90% N_2_/10% H_2_ atmosphere in an anaerobic chamber (Concept 400; Ruskinn Technology). An overnight grown culture (12 h old) was split into two parallels per 240 ml, and the antibiotic chloramphenicol (30 μg ml^–1^) was added to one of the parallels. This point was set as time point zero (t0). After 1 h of incubation, a sample was withdrawn from both untreated (t1) and chloramphenicol-treated (t1_CH) cultures.

### RNA Isolation and Reverse Transcription

Samples of the culture broth for the RNA isolation were centrifuged (13,000 *g*, 2 min, 4°C). The pellets were then washed with ice-cold distilled water and resuspended and diluted to reach optical density (measured at 600 nm) of approximately 1.0. Next, 3 ml of the suspension with the OD_600_ ≈ 1.0 sample were centrifuged, and the cell pellet was immediately stored at −80°C for subsequent RNA isolation. Frozen samples were thawed on ice, and the total RNA was isolated using the High Pure RNA Isolation Kit (Roche Life Science, Basel, Switzerland), according to the manufacturer’s instructions. The quality and concentration of the RNA samples were checked on a nanodrop machine (Thermo Fisher Scientific, Waltham, MA, United States).

Reverse transcription of RNA samples was performed with the Transcriptor First Strand cDNA Synthesis Kit (Roche Life Science, Basel, Switzerland), according to the manufacturer’s instructions.

### Quantitative Real-Time PCR

All RT-qPCR analyses were performed in QuantStudio 5 instrument (ThermoFisher Scientific, Waltham, MA, United States) using the 5 × HOT FIREPol EvaGreen qPCR Mix Plus (ROX) (Solis BioDyne, Tartu, Estonia). The reaction mix (20 μl) was prepared according to the manufacturer’s instructions, using each primer (see Table 2) in the final concentration of 200 nM. The cycling conditions were set according to the manufacturer’s instructions, using a primer annealing temperature of 61°C. Primer specificity was confirmed through melt curve analysis after the cycling stage (95°C for 15 min, 61°C for 1 min, 95°C for 1 s). All RT-qPCR analyses were performed in triplicates, and the absence of contamination was confirmed by running no-template and no-RT controls.

**TABLE 2:** Sequences of primers used for quantitative real-time PCR.

Target gene	Forward primer	Reverse primer
*zmp* (X276_20795)	TGCATCAACAA GGGCTTTGAA	TATGTCATTG CTGCTGCGTC
*rpoB1* (X276_25940)	ACGAAGCTAG GACCAGAGGA	TGGCGTTACC TTTCCAACCA
*rsmB* (X276_20790)	AGTGCACCTG GTGGTAAAACT	CCAAGCCCTG AACAAGGAAC
*greA* (X276_26205)	GCAGAAGCGGA CCCTATGAA	TCCGTCTGGA ACTGTTACCTC
*rpoB2* (X276_25935)	AAGAGCGCC AAGAGAATGGT	ACAGCCATTT GGTCCCCATC
*topB2* (X276_03960)	TATTTAGCG CAGCCCCCATT	TCTGCTTTTG CCGCATCTTC
*rimO* (X276_20450)	GCTGCTAAAA TGGATGGGCA	GAGCCATTTCAT AGCTTCTTCCA

Polymerase chain reaction efficiency was determined for each sample using a 5x serial dilution of cDNA samples (5x, 25x, 125x, and 625x), ranging between 94 and 110% for all samples with the correlation coefficient *R*^2^ > 0.99. For each gene in each sample, the average *C*_*q*_ value from the dilution interval 5–625x was used for further data analysis.

### RT-qPCR Data Analysis

Four different algorithms or statistical methods were used for the evaluation of the stability of gene expression. These included NormFinder (Andersen et al., 2004), RefFinder (Xie et al., 2012), CV analysis (Boda et al., 2009) and Pairwise ΔCt method (Silver et al., 2006).

## Results

### RNA-Seq Data Analysis

In order to identify putative candidates for reference genes, we evaluated 5,442 predicted genes of *C. beijerinckii* and applied several filtering steps to reveal genes with stable expression indifferent in time and under different conditions (see section “Materials and Methods”).

First, we removed 4,370 genes based on the results from the differential expression analysis. Next, we eliminated genes according to their mean TPM and the number of candidates dropped from 1,072 to 448 genes. Finally, we applied filtering step based on the CV of TPM and only 160 genes remained as candidates for reference genes (see Supplementary Material 2).

In the next step, we narrowed the list of the candidate genes by considering their biological function as well as the fact that the genes with the same function were reported to be putative reference genes and/or used as reference genes in RT-qPCR experiments in other publications (see Table 3).

**TABLE 3:** Candidate reference genes of *C. beijerinckii* NRRL B-598.

Gene annotation	Abbr.	Locus tag	References
Zinc metallopeptidase	*zmp*	X276_20795	Chi et al., 2016
DNA-directed RNA polymerase subunit beta	*rpoB1*	X276_25940	Metcalf et al., 2010; Kirk et al., 2014; Wang et al., 2014; Gomes et al., 2018
16S rRNA [cytosine(967)-C(5)]-methyltransferase RsmB	*rsmB*	X276_20790	Metcalf et al., 2010; Peng et al., 2014; Liu et al., 2017; Delorenzo and Moon, 2018
Transcription elongation factor GreA	*greA*	X276_26205	Song et al., 2019
DNA-directed RNA polymerase subunit beta’	*rpoB2*	X276_25935	Metcalf et al., 2010; Kirk et al., 2014; Wang et al., 2014; Gomes et al., 2018
Type IIA DNA topoisomerase subunit B	*topB2*	X276_03960	Metcalf et al., 2010; Carvalho et al., 2014
30S ribosomal protein S12 methylthiotransferase RimO	*rimO*	X276_20450	Song et al., 2019

*These genes were selected based on the RNA-Seq data, their biological function, and the literature review.*

Calculated CV values of seven selected genes based on TPM values ranging from 18.7 to 29.4%. The most stable genes were *rsmB*, *zmp*, and *rimO* (see Table 4).

**TABLE 4:** Coefficient of variation of transcript per million (TPM) values from both count tables (uniquely mapped reads and multi-mapping reads).

Gene abbreviation	Uniquely mapped reads	Multimapping reads
	
	CV (%)	CV (%)
*rsmB*	18.7	18.8
*zmp*	19.3	20.4
*rimO*	21.9	22.2
*greA*	22.7	22.6
*rpoB2*	22.8	22.8
*rpoB1*	25.1	24.9
*topB2*	29.4	28.9

Finally, we performed GO enrichment analysis of the final set of genes using all genomic loci as the gene universe to describe their functionality. We found 22 biological process (BP) GO terms (see Table 5) and 12 molecular function (MF) GO terms (see Table 6) that were significantly enriched (*p*-value < 0.05, Fisher’s exact test). From BP terms, the most significant terms related to “nucleic acid metabolic process,” “macromolecule metabolic process” or “cellular nitrogen compound metabolic process.” The most common term for MF GO terms was “nucleic acid binding.”

**TABLE 5:** Biological process (BP) gene ontology (GO) enrichment results.

GO.ID	Term	Annotated	Significant	Expected	Classic Fisher
GO:0090304	Nucleic acid metabolic process	710	6	1.41	0.00016
GO:0006139	Nucleobase-containing compound metabolic process	885	6	1.75	0.00062
GO:0016070	RNA metabolic process	505	5	1.00	0.00066
GO:0044260	Cellular macromolecule metabolic process	983	6	1.95	0.00116
GO:0006725	Cellular aromatic compound metabolic process	993	6	1.97	0.00123
GO:0010467	Gene expression	576	5	1.14	0.00124
GO:0046483	Heterocycle metabolic process	996	6	1.97	0.00126
GO:1901360	Organic cyclic compound metabolic process	1,014	6	2.01	0.00140
GO:0018197	Peptidyl-aspartic acid modification	1	1	0.00	0.00198
GO:0018339	Peptidyl-L-beta-methylthioaspartic acid biosynthetic process from peptidyl-aspartic acid	1	1	0.00	0.00198
GO:0034641	Cellular nitrogen compound metabolic process	1,084	6	2.15	0.00209
GO:0009451	RNA modification	44	2	0.09	0.00299
GO:0006351	Transcription, DNA-templated	390	4	0.77	0.00329
GO:0097659	Nucleic acid-templated transcription	391	4	0.78	0.00332
GO:0032774	RNA biosynthetic process	394	4	0.78	0.00342
GO:0043170	Macromolecule metabolic process	1,198	6	2.37	0.00381
GO:0018198	Peptidyl-cysteine modification	2	1	0.00	0.00396
GO:0034470	ncRNA processing	55	2	0.11	0.00464
GO:0006354	DNA-templated transcription, elongation	3	1	0.01	0.00594
GO:0032784	Regulation of DNA-templated transcription	3	1	0.01	0.00594
GO:0006396	RNA processing	63	2	0.12	0.00606
GO:0034654	Nucleobase-containing compound biosynthesis	522	4	1.03	0.00979

**TABLE 6:** Molecular function (MF) gene ontology (GO) enrichment results.

GO.ID	Term	Annotated	Significant	Expected	Classic Fisher
GO:0003899	DNA-directed 5′–3′ RNA polymerase activity	6	2	0.01	3.6e-05
GO:0034062	5′–3′ RNA polymerase activity	7	2	0.01	5.0e-05
GO:0097747	RNA polymerase activity	7	2	0.01	5.0e-05
GO:0140098	Catalytic activity, acting on RNA	97	3	0.16	0.00037
GO:0103039	Protein methylthiotransferase activity	1	1	0.00	0.00169
GO:0003676	Nucleic acid binding	803	5	1.36	0.00285
GO:0019899	Enzyme binding	2	1	0.00	0.00338
GO:0035596	Methylthiotransferase activity	2	1	0.00	0.00338
GO:0070063	RNA polymerase binding	2	1	0.00	0.00338
GO:0016779	Nucleotidyltransferase activity	65	2	0.11	0.00472
GO:0050497	Transferase activity, transferring alkylthio groups	3	1	0.01	0.00506
GO:0003918	DNA topoisomerase type II (double strand cut, ATP-hydrolyzing) activity	5	1	0.01	0.00842

### Analysis of Expression Stability Based on RT-qPCR Data

**FIGURE 1:**
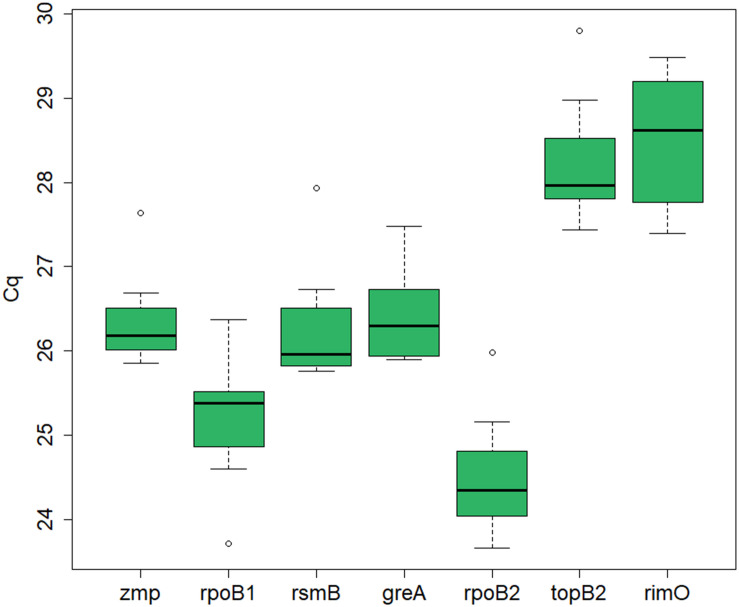
RT-qPCR Cq values for the candidate reference genes in *C. beijerinckii* NRRL B-598. Depicted is the median Cq value calculated for each candidate reference gene across all samples. The boxes indicate the 25th and 75th percentiles and the error bars indicate the maximum and minimum values gained across the samples for each gene.

The expression levels, represented by the *C*_*q*_ values, of the seven candidate reference genes (*zmp*, *rpoB1*, *rsmB*, *greA*, *rpoB2*, *topB2*, and *rimO*) were assessed by RT-qPCR in seven cultivation samples. The samples were acquired in experiments with different cultivation conditions (standard, i.e., untreated culture, butanol-treated culture, and chloramphenicol-treated culture) and at different time points. The mean *C*_*q*_ values ranged from 20.2 to 33.3 for different dilutions and different genes in different samples (see Supplementary Material 1). An average *C*_*q*_ value was calculated for each gene in each sample across all dilutions, which was used for further analysis of expression stability. The median *C*_*q*_ for the different candidate genes ranged from 24.3 to 28.6, indicating that the expression levels of the different genes were not dramatically different (see Figure 1).

**FIGURE 2:**
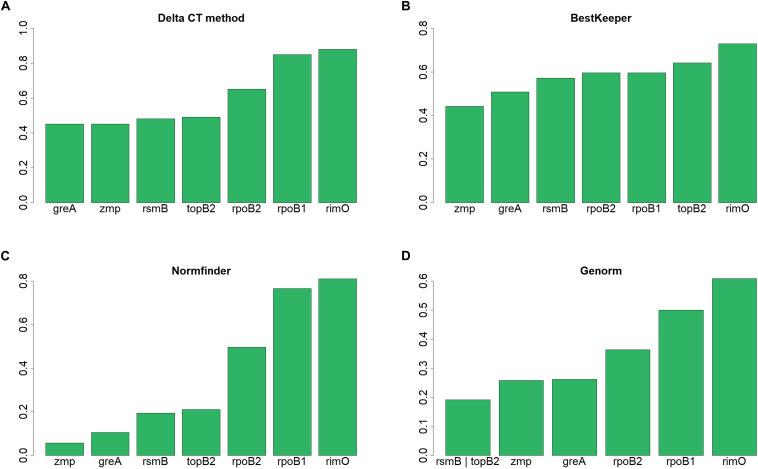
Expression stability of candidate reference genes in *C. beijerinckii* NRRL B-598 assessed by RefFinder tools. **(A)** Delta CT method, **(B)** BestKeeper, **(C)** Normfinder, and **(D)** Genorm.

According to NormFinder, CV and Pairwise ΔCt analyses, the genes with the most stable expression were *zmp*, *greA*, and *rpoB2* (see Table 7 and Supplementary Material 1). Using NormFinder (Andersen et al., 2004), the samples were gathered into three groups with respect to the three different cultivation experiments from which they originated. According to RefFinder, including multiple analytical tools (Delta CT, BestKeeper, Normfinder, and Genorm), the comprehensive ranking of the genes with respect to expression stability was *zmp*, *greA*, *rsmB*, *topB2*, *rpoB2*, *rpoB1*, and *rimO*, with *zmp* and *greA* appearing as the best combination based on three out of the four tools (see Figure 2). Using the RefFinder tools, the samples were not gathered into groups. Taken together, *zmp* and *greA* appear to be the most suitable combination of genes that can be used for normalization of RT-qPCR experiments under tested conditions in *C. beijerinckii* NRRL B-598.

**TABLE 7:** Expression stability of candidate reference genes in *C. beijerinckii* NRRL B-598 assessed by different analytical tools.

Ranking	NormFinder (Andersen et al., 2004)		Coefficient of variation analysis (Boda et al., 2009)		Pairwise Δ Ct method (Silver et al., 2006)	
	Gene	Stability value	Gene	CV*^1^ (%)	Gene	Average SD*^2^
1	*zmp*	0.129	*zmp*	30.9	*rpoB2*	0.455
2	*greA*	0.283	*greA*	38.8	*zmp*	0.460
3	*rpoB2*	0.357	*topB2*	43.1	*topB2*	0.481
4	*rsmB*	0.379	*rpoB2*	46.3	*rpoB1*	0.488
5	*topB2*	0.403	*rsmB*	48.6	*rsmB*	0.650
6	*rpoB1*	0.495	*rpoB1*	63.7	*greA*	0.846
7	*rimO*	0.630	*rimO*	70.3	*rimO*	0.880
Best combination	*zmp* + *greA*		*zmp* + *greA*		*rpoB2* + *zmp*	

*^1^coefficient of variation and *^2^standard deviation.

## Discussion

Normalization by reference genes is required for a precise analysis of gene expression by RT-qPCR. The choice of the reference genes, however, should not solely rely on the gold standard used for the particular cell type, such as 16S rRNA in the case of bacteria (Rocha et al., 2015), but should always be systematically and experimentally validated (Bustin et al., 2009). Moreover, according to the MIQE guidelines (Bustin et al., 2009), normalization against more than one reference gene is preferred.

In the case of *C. beijerinckii* NCIMB 8,052, a list of putative reference genes based on the analysis of transcriptomic data was previously published (Wang et al., 2011), and one of the candidate genes (peptidase T, Cbei_2428) was subsequently used as a reference for RT-qPCR experiments (Wang et al., 2012). However, completely different set of putative reference genes was generated for closely-related *C. beijerinckii* NRRL B-598 during our analysis of RNA-Seq data of the strain. Only Cbei_1214 was matched by X276_20450 in *C. beijerinckii* NRRL B-598, and X276_14515, a homolog of Cbei_2428 used in Wang et al. (2012) study, has not found to be a suitable reference gene in our case (see Supplementary Material 2 and section “Discussion” below). We investigated the gene expression stability in *C. beijerinckii* NRRL B-598 by evaluating both transcriptomic and RT-qPCR data. Based on these, we suggest a set of appropriate reference genes regarding the given set of tested conditions (see section “Materials and Methods”).

Identification of candidate reference genes in *C. beijerinckii* was performed by bioinformatics analysis of our previously published RNA-Seq data (Sedlar et al., 2018, 2019; Patakova et al., 2019). All data were processed again with up-to-date software and packages and we have introduced a new approach to the selection of reference genes based on RNA-Seq data. Pre-processing of our RNA-Seq data remained the same until the counting of mapped reads. Here, we decided to create two different count tables with two counting options. The first counting method is strict counting of unique reads mapped to the genomic loci and the second method also considers multi-mapping reads. Contribution of all multi-mapping reads were split equally among all genomic objects they mapped to. Therefore, the number of reads in the sample remained the same. Multi-mapping reads present a problem for downstream analyses as they reduce sensitivity (Canzar et al., 2016). Although several strategies and specialized tools were proposed to count multi-mapping reads (Roberts et al., 2013; Zytnicki, 2017), for our purposes, specificity was the principal issue. By selecting only genes where differential expression was not detected using uniquely neither multi mapped reads, we reduced the possibility of a type I error and, therefore, improved specificity. We are aware that this approach led to a higher possibility of type II error and, thus, to lower sensitivity. However, this would be problem only for following differential analysis aiming at finding differentially expressed genes. In our case, the aim was to completely reduce ambiguities. Next, we decided to calculate TPM values from obtained count tables, as TPM corrects differences in library sizes and gene lengths and enables comparisons among samples (Wagner et al., 2012).

A selection of housekeeping genes was performed by evaluation of the counts of insignificant changes in expression obtained from differential expression analysis, mean TPM values, and the CV of TPM values. First, filtering based on the results of the differential expression analysis, which we used to find genes with stable expression and minimal regulation, removed genes that were regulated in more than 25% of possible time-points pairs. In the next step, we eliminated genes with low mean TPM values as we were looking for genes with moderate stable expression. Low TPM values can result from technical noise in the data even after filtration. In the last step, we focused on the filtration of genes with a gradual increase or decrease in expression, which would not be discovered by differential expression analysis. For filtering, we used the CV of TPM values that can reflect those slow changes in expression and is often used in other studies geared toward the selection of reference genes (Carvalho et al., 2014; Hu et al., 2016; Pombo et al., 2017). Only 16 out of 160 genes identified by the RNA-Seq data analysis corresponded to the previously identified 177 housekeeping genes in *C. beijerinckii* NCIMB 8,052 (Wang et al., 2011) (see Supplementary Material 2). While this might suggest that the reference genes identified in this study are not utilizable for other *C. beijerinckii* strains, we believe this is rather a matter of the simplified methodology used by Wang et al. (2011). In their study, the identification of HKGs was only a supplementary result and calculating CV from RPKM values appeared to be inappropriate (Wagner et al., 2012). Moreover, identification of HKG based solely on the RNA-Seq data is less specific, see section “Discussion” below.

Final selection of the seven candidate reference genes (see Table 3) for RT-qPCR experiments was summarized by a GO enrichment analysis. GO enrichment analysis of biological processes revealed 22 enriched terms at significance level α = 0.05 of the Fisher’s exact test (see Table 5) and most of the enriched terms related to “nucleic acid metabolic process” (GO:0090304) and “RNA metabolic process” (GO:0016700) terms, which correspond with terms identified in other bacterial strains (Rocha et al., 2015). MF GO enrichment analysis revealed ten enriched terms and the most common term “nucleic acid binding” (GO:0003676) corresponds with identified biological processes (see Table 6).

Based on the RT-qPCR experiments, *zmp* (zinc metallopeptidase), *greA* (transcription elongation factor GreA), and *rsmB* (16S rRNA (cytosine(967)-C(5))-methyltransferase RsmB) were the most stably expressed genes (see Table 7 and Figure 2). In pathogenic *Clostridium* species, the zinc metalloproteases act like neurotoxins (Breidenbach and Brunger, 2004) or are involved in the degradation of extracellular substrates (Cafardi et al., 2013). The transcription factor GreA is evolutionarily conserved and widely distributed in prokaryotes (Marr and Roberts, 2000). The 16S rRNA-methyltransferases ensure methylation of 16S rRNA and in Gram-negative bacteria are involved in resistance to aminoglycosides (Lioy et al., 2014). In Gram-positive bacteria, 16S rRNA-methyltransferases was required for resistance to tetracycline antibiotics in the case of *Streptococcus pneumoniae* (Lupien et al., 2015), and to oxidative stress in the case of *Staphylococcus aureus* (Kyuma et al., 2015). The *zmp* gene figured amongst the best candidate genes in *Vigna angularis* (Chi et al., 2016), the transcription elongation factor gene in *Vernicia fordii* Hemsl. (Han et al., 2012), and the 16S rRNA methyltransferase gene in *Rhodococcus opacus* (Delorenzo and Moon, 2018). Generally, the three most commonly tested and validated bacterial reference genes are 16S ribosomal RNA, DNA gyrase A, and recombinase A (Rocha et al., 2015). In our case, the genes of 16S rRNA and recombinase were not within the list of the 160 candidate genes after the bioinformatics analysis of the transcriptomic data (see Supplementary Material 2). This was expected for the 16S rRNA gene as wet-lab ribodepletion was performed before sequencing and the remaining contamination was removed *in silico* with SortMeRNA. The DNA topoisomerase (gyrase) subunit was within the list, though did not rank amongst the genes with the most stable expression, according to the RT-qPCR results. In *C. beijerinckii* NCIMB 8,052, gene encoding peptidase T was chosen as a reference gene (Wang et al., 2012). In *C. difficile*, the genes of 16S rRNA, adenylate kinase, and 30S ribosomal protein S10 displayed the most stable expression within eight tested candidate genes (Metcalf et al., 2010). Nevertheless, the ranking differed for three different *C. difficile* strains tested (Metcalf et al., 2010), confirming the need for a careful selection of reference genes for each species (Bustin et al., 2009). The 16S rRNA gene was also chosen as a reference in *C. botulinum* (Kirk et al., 2014). The most suitable genes for normalization of gene expression in *C. ljungdahlii* were genes of gyrase subunit A, transcriptional termination factor, and formate-tetrahydrofolate ligase (Liu et al., 2013). Within the set of the tested candidate genes, the bacterial standards of 16S rRNA and recombinase A genes (Rocha et al., 2015) displayed the least stable expression in *C. ljungdahlii* (Liu et al., 2013).

The need for the validation of genes selected using only RNA-Seq data is supported by the comparison of the coefficients of variation calculated from C_*q*_ (see Table 7) and the TPM values (see Table 4). Although genes *zmp* and *greA* maintain a high rating in both statistics, suggesting that rankings can in some cases correlate, results for *rsmB* and *rimO* genes tell otherwise. The *rsmB* gene was the most stable according to the RNA-Seq data, yet RT-qPCR experiments show that the other three respectively, four genes were more suitable as candidate genes (see Table 7). In the case of the *rimO* gene, which was the third most stable gene according to the TPM values, it dropped to the last place after experimental validation. Moreover, the average CV of C_*q*_ values was more than two times higher than the average CV of TPM values (2.14 × for uniquely mapped and 2.13 × for multi mapped reads) (see Tables 4, 7), indicating that the RNA-Seq provides only less specific results selecting a wider range of genes on a given CV threshold value. These results suggest that the selection of reference genes cannot be performed by RNA-Seq data analysis alone, yet it can be used for the compilation of candidate genes list. However, validation by RT-qPCR experiments is always needed.

## Conclusion

We identified and validated a novel set of reference genes of *C. beijerinckii* NRRL B-598. We selected 160 candidate reference genes based on analysis of all currently available RNA-Seq data for the strain covering several different experimental conditions. Selection of seven genes (*zmp*, *rpoB1*, *rsmB*, *greA*, *rpoB2*, *topB2*, and *rimO*) was summarized by GO enrichment analysis and further validated by RT-qPCR assays and statistical testing by four statistical algorithms (NormFinder, RefFinder, CV analysis, and Pairwise ΔCt method). The analysis ranked seven genes by their expression stability, presenting zinc metallopeptidase (*zmp*) and transcription elongation factor GreA (*greA*) as an appropriate set of reference genes regarding tested set of conditions. Our results can be helpful for selection of reference genes in other *C. beijerinckii* strains, and our methodology suggests that RNA-Seq data can be used for identification of novel reference genes, but their validation by RT-qPCR experiments is always needed.

## Data Availability Statement

Publicly available datasets were analyzed in this study. This data can be found here: all computational analyses using sequencing data in this work are done against the reference sequence deposited at DDBJ/ENA/GenBank under the accession number CP011966.3. RNA-Seq data used in this study are available from NCBI SRA database under the accession numbers SRR6375604, SRR10556738–SRR10556761 and SRR10569082–SRR10569093.

## Author Contributions

KJ: conceptualization, methodology, formal analysis, data curation, visualization, and writing-original draft preparation. HS: conceptualization, methodology, validation, formal analysis, visualization, and writing-original draft preparation. JK: writing-original draft preparation. MV: validation and investigation. BB: conceptualization and investigation. PP and IP: supervision, resources, and funding. KS: conceptualization, methodology, formal analysis, and writing-original draft preparation. All authors contributed to the article and approved the submitted version.

## Conflict of Interest

The authors declare that the research was conducted in the absence of any commercial or financial relationships that could be construed as a potential conflict of interest.
